# Selections and Abstracts

**Published:** 1913-02

**Authors:** 


### SELECTIONS AND ABSTRACTS

### OCULAR HEADACHE

By Aaron Brav, M. D., Philadelphia

Headache is the most common ill that human flesh is heir to. Society at large is subject to its preying at some period of life. It is no respecter of person. Rich and poor alike, men and women, young and old, the learned and the ignorant, all are occasional sufferers; some more, some less. No occupation or walk of life is immune, although some vocations may be considered as predisposing factors. The man who guides the destiny of the nation, the man who controls the money market, the genius and the inventor, the professional as well as the business man the artist and the musician, the capitalist as well as the laborer is found to suffer from it.

Headache is not a disease, but a symptom of some abnormal condition. There are different kinds of headaches; some are localized, while others are diffuse. Headaches may be perceived as a sharp pain, excruciating in character it may be, on the other hand only a dull aching pain. It may be of a throbbing character or of a pressure nature. As to locality, it may be frontal, temporal, occipital, or radiating from these points to others parts. The locality and type of the pain does not always indicate with sufficient accuracy the cause of the headace. Occasionally the headache is confined to one side of the head when we speak of it as hemicrania. Headache may be the only symptom, the only complaint of the patient; he may be otherwise in perfect good health. On the other hand, it may be one of a series of symptoms pointing to some diseased condition. The importance of this condition can be surmised when one can say with a degress of certainty that there is not a home in which this condition does not constitute a continuous source of misery to one or several members of the family.

Headache may be steady, continuous, a daily visitation to the patient, bringing misery unto him, or it may be periodical, although both may have one causal element. This variation in the degree and type, where one causal element is responsible for the phenomenon, can be explained only on the ground of differences in the resisting power of the individual, his mode of living, as well as his occupation. Women are common sufferers from headache.

Headaches may be due to various etiological factors. They may accompany the various acute infections, as well as various acute diseases of the eye. We shall, however, in this paper consider only the chronic form of headaches. The responsible cause for these headaches is in about eighty per cent. of the cases some error of refraction, or eye strain. In fact, it may be stated as an axiom that headache of whatever type occurring in an otherwise healthy individual, and not associated with any of the acute infectious diseases, may with rare exception be considered an ocular headache. By the term ocular headache is meant one that is caused by some error of refraction or muscular imbalance and therefore amenable to prompt cure. As stated above, headache may be the only symptom resulting from eye strain or it may be associated with other symptoms. These other symptoms may be the direct result of the error of refraction, or they may be the sequalæ of the primary symptoms, namely, the headache. Headache is only a symptom, but it may be ultimately responsible for many other symptoms of nervous irritability. In fact, the continuous sufferers from headaches manifest various nervous symptoms. Continuous headaches, even of the intermittent type, make a deleterious impression upon the entire body. Those who are unfortunate enough to suffer from headache for any length of time will best be able to testify to the baneful effect it has upon the general condition. One may even indulge in the supposition that nearly all of the symptoms observed in eye strain are merely secondary to the primary headache. Headache is the direct result in about eighty per cent. of cases of some ocular anomaly, refractive or muscular, and if neglected and permitted to grow into a habit it so saps the vitality of the patient that it soon gives rise to other symptoms.

Continuous headache brings misery into the home, deprives the patients of all pleasures, and ultimately makes nervous wrecks of them. It must be remembered after all that the pain is referred to the brain, which is the most important organ controlling all our emotions, and is the center of our nerve force, that any continuous and prolonged pain in that part of our body will disturb our mental equilibrium and produce a chain of symptoms which may simulate some serious nervous disease. Thus the patient with habitual headaches becomes irritable, restless, may occasionally suffer from insomnia, disturbances of memory, lack of concentration, dizziness and vertigo, gastrointestinal disturbances; in fact, headache may give rise to a chain of symptoms simulating some very serious condition of the body. Patients with a weak bodily condition may, as a result of prolonged suffering from headaches, become confirmed neurasthenics, while as a matter of fact the underlying disturbing causal element was simply an error of refraction that was either not recognized or if recognized not well corrected.

It may be laid down as an axiom that eye strain is the most prolific source of headache. Any error of refraction may give rise to eye strain and cause headaches, but it is found mostly in the astigmatic type, simple, compound, or mixed. It is interesting to note that in the majority of cases the eyes are free from pain and vision as tested by Snellen's card is normal. The reflex symptoms of eye strain seem to be in inverse ratio to the ocular symptoms. Normal vision does not mean a normal dioptric apparatus, for normal vision may be obtained with high degrees of error of refraction, such as hyperopia. But this vision is obtained by means of an extraordinary expenditure of nerve force exerted upon the ciliary muscles. The ciliary muscles, in response to this extra nerve stimulus, have to do an extra amount of work, and this overexertion causes reflexly the headache. We must remember that the eye is the most overworked organ in the body, beginning its work as soon as we awake in the morning and continuing until late at night when we fall asleep. There is no rest even at the noon hour; so that the external and internal ocular muscles are continuously at work for about sixteen hours every day. When to this arduous labor is added the muscular and nervous effort necessary to overcome any anomaly of refraction and muscular imbalance, we can readily appreciate why such overwork should be followed by headaches.

### TREATMENT

It is our experience that patients suffering from frequent and prolonged headache soon learn how to treat themselves, although to their own detriment. They acquire a wonderful versatility in the various preparations on the market. All headache preparations in powder or tablet form have for their basic constitutent some of the coaltar products, which if taken in sufficient quantity give temporary relief. The effect of the drug is soon transferred, however, to the heart and the nervous system, where it leaves an everlasting impression and only tends to make the patient more miserable. The chief danger in all the so-called headache medicines is their temporary sedative effect, which lulls the patient into acquiring the drug habit, that will untimately make a nervous wreck of him. The public press now and then reports a case of death which resulted directly from the prolonged use of such headache cure; this is, however, not very frequent. The majority of the headache powder patients suffer in their mental and psychophysical make up, reducing their intrinsic vitality, and weakening their cardiac musculature, which can only show itself when cardiac and nerve force is most necessary—to overcome some acute disease. Even the moral fibre of these drug habitues is dulled, blunting their power of reason as well as their creative faculty, impairing their intellect, so that they are useless in the performance of tasks that require a high degree of intelligence.

There is sufficient reason to think that the laity has learned this mode of treating headaches from the medical profession, and it is certainly a fact that most headache cures sold in the market behind the counter are merely copies of some prescription by some known or unknown physician. It is true that theoretically we classify headache into different categories, ascribing to each some definite causal element and advise to find the cause before any treatment is attempted, but in reality, when confronted with a patient suffering from headache, what is the usual mode of treatment adopted by the average physician? Are we in reality not doing the same thing that some of the patients do themselves? Do not we have recourse in ninety per cent. of cases to the dangerous coal tar products? Phenacetine, antipyrine, acetanilide, and others are the usual ingredients, probably in some combination with some other drug that is handed out to the patient. The only difference between the self doctored and the professional prescription is that while under professional care the danger of overuse is somewhat lessened. Not very often does the doctor think of an error of refraction, of eye strain that requires correction, which is the offending cause, and at best he directs his patient to go to the hospital, or the patient of his own accord consults an optician. Optical adjustment, however, requires very careful attention, which the routine work of the hospital cannot supply, while the optician is absolutely deficient in his ability to cope with the situation. Not only is it essential to correct any existing error of refraction, but it is also of utmost importance that the equilibrium of the muscular apparatus be reestablished wherever any disturbance exists. It is my personal conviction, based upon the study of several thousand cases, that fully eighty per cent. of chronic sufferers from headache can attribute their trouble to some faulty optical condition that can be corrected by the man who is efficient in that work.

It is well here to mention that some of these cases need some medical aid even after the optical error has been corrected. It is true that the cause of the headache has been removed by the properly prescribed and adjusted lenses, but the sequalæ of a protracted headache are still present and must engage our attention. Hygenic and dietetic measures, as well as a tonic treatment, will usually in a short time readjust all the possible changes that resulted from the depressing effect of a prolonged and protracted headache. Often the muscular disturbance must be treated by prism exercises. I have seen many patients whose refractive error has carefully been adjusted by some prominent oculist, yet this has failed to give relief simply because some disturbance in the external muscles has been neglected. Children very often during school life suffer from headaches, and their suffering is continued merely because parents often object to their wearing glasses. Physicians should insist in these cases upon optical aid, and the parents may be assured that this optical aid will be necessary only while the child attends school. To permit a child suffering from headache to attend school without optical aid is nothing short of criminal, for soon these children become anemic and nervous, which prevents them from concentrating their minds upon the subjects they have to master, and usually they constitute a large number of the so-called failures, not because of an inherent mental deficiency, but because of the ocular trouble which partly disables them.

A few words about the optician. A great deal of harm is done to this class of sufferers by the optician, who still has a hold upon the uneducated public, and constitutes a great number in any community. Most of these men are not only ignorant of the basic principles of physiological optics, but have not even received a common school education, and a considerable number of them are positively void of any sense of honor in their dealing with patients suffering from some ocular condition. To their ignorance must be added a dishonest desire to defraud the public. Knowing that there are no laws in this country sufficient to protect the public against all kinds of medical fakes, nor any law to prevent any one from calling himself a doctor, they assume that title and ply their trade under the self assumed title in private offices with the sole intention of defrauding an innocent public. The public cannot differentiate them from the educated oculist. The public is under the impression that the title doctor can be used only by a man who had to go through a regular college course and had to qualify before a state board of examiners and legally register in the respective locality in which he resides. Therefore, they place confidence in these men, consult them for ocular troubles and eye strain. As a matter of fact, however, these men are mere charlatans using the title doctor to defraud the public. I charge them with dishonesty. If they had any sense of decency and attended to their business honestly, why would they use the title doctor, for which they neither toiled nor labored? Their pretension to the title convicts them of fraud and dishonesty. Some day I suppose the laws of the land will prevent the parasites from using the title doctor for selfish fradulent purposes; until then we are helpless and the community at large must suffer the consequences.

I a maware that there are other causes for headache that the practitioner has to search for, but I am in this paper concerned only with headaches that are ocular in origin and of a chronic nature, and they afflict the largest class of sufferers and yet are most easily amenable to treatment. All patients suffering from headaches should be carefully "refracted," and in eighty per cent. of cases a proper pair of glasses will bring about a cure. After all, a properly prescribed pair of glasses is the best and least harmful therapeutic agent that we have in the treatment of headaches.—*New York Medical Journal.*

---

### THE TRANSMISSION OF POLIOMYELITIS

The recent epidemic of poliomyelitis in Sweden throws some ligt.on the transmission of the disease.

The epidemic broke out simultaneously in four widely separated districts in the beginning of April, 1911, and radiated in various directions from these centers. The epidemic showed a tendency to spare areas previously attacked, but the neighboring districts seemed predisposed to the disease. It is possible that acquired immunity may be inherited, since the children born in previously affected districts in the interval following the last epidemic, were more resistant to the diseases than the children in the previously unaffected districts.

The biennial periodicity observed in other countries has not been noted in Sweden. The topographical distribution shows some curious features. Marshy districts remained free. Frost and snow checked the outbreak in some places. Sanitation and hygiene evidently played no part in preventing the spreading infection, as the disease showed a tendency to attack small isolated villages in woodland districts, while sparing large industrial centers where overcrowding and unhygienic conditions prevail. In some localities the infection could be traced along the railroads and highways. Infection could not be traced to milk or meat during this epidemic, but cannot be safely excluded. Wickmann established that infection fomites took place in at least one case in the epidemic of 1905. In the first case which then developed in Stockholm, the infection was carried through notes and drawings on which a young draughtsman had been working during convalescence. That the virus may be transmitted by a healthy carrier over great distances was illustrated during the first epidemic in Westphalia, which broke out after a Swedish engineer residing there returned from Sweden, where a member of his family was suffering from the disease.

Indirect transmission by insects is, according to the Swedish investigators, only of importance in house epidemics, especially as regards blood-sucking insects. They failed to induce experimental poliomeyelitis with saline infusion of flies caught in the epidemic wards by the patients.

Clark and Howard, of the Rockefeller Institute, have, however, demonstrated that the virus is still potent after having been carried on the body of the house fly for several days. Inoculations of filtrates from the bodies of flies fed on material infected with the virus were successful.

Rosenau and Brues have gone one step further and suggest that poliomyelitis, like filariasis, malaria and yellow fever, is transferred from one subject to another through an intermediate host, and that the causative agent passes through a definite cyle of evolution within the body of the intermediate host. There is strong evidence in favor of this theory. Rosenau and

Brues have thus "transferred the virus of poliomyelitis from monkey to monkey through the bite of the stable fly, *Stomoxys calcitrans.*" The Massachusetts State Board of Health have pointed out that the disease shows a predilection for localities where farm animals are kept.

The now generally accepted theory that the causative agent enters through the upper air passages, is borne out by the investigations of Patterson, Kling and Wernstedt. These authorities found the secretions from the nasopharynx infective in about four-fifths of the manifest cases and in a large percentage of absortive cases and healthy contacts. Whenever the intestinal secretion is infective it is due to swallowing of infected nasopharyngeal secretion. It has been proved by Flexner that the virus withstands the antiseptic action of the gastric juice.

The average period of infectivity after the acute stage, is four months, seven months being the maximum time reported. The virulence is so rapidly attenuated after the acute stage has passed, that a quarantine of four weeks, from the time of onset, is sufficient. The contagiousness is at its height during the incubation period, and is also greater in the absortive than in the manifest cases, but as the absortive cases are five times as numerous as the manifest ones, prophylaxis through effective isolation is well neigh. impossible.—*La Tribune Medicale.*

---

### SURGERY FOR CRIMINAL TENDENCIES

An interesting after-development in a surgical case which attracted much attention about four years ago has just occurred. A prisoner serving a long sentence in the prison at Dannemora, N. Y., was pardoned by Gov. White on representations which seemed to make it clear that he had been cured of his criminal tendencies by a surgical operation. The prisoner was comparatively young, but from boyhood had been noted for his tendencies to appropriate the property of others, not only by taking, but by clever frauds and by forgery. When given this long term he had carefully studied his own case, it was said, and had found that at the age of about 14 he had been struck on the head with a fence-picket. The result was a fracture of the skull for which the subsequent prisoner had to stay for a long time in the hospital. Before this, according to the story as detailed by the prisoner, he had been an exemplary youth. After this he became morose, sullen and a theif, and stole from every one. Becoming convinced that his criminal tendencies were due to this injury to his head, he sent for the family physician who had treated him, not for the injury, but some years afterward.

He set his case clearly before the physicians whom the family physician called in consultation, and finally convinced them that perhaps there was something in his story. He had been reading books on criminology and was quite sure that the root of the evil was a physical defect and not a moral perversion in his own case. He represented that often when he took things he had no need for them; that even the large forgery for which he was under sentence had been committed at a time when he had not particular need for money, and that there must be something else besides an ordinary tendency to thieving in his case. An examination of his skull showed an old thickening underneath the scalp at the point where he had been injured. Apparently this thickening might produce some pressure on the brain, and since he pleaded it seemed worth while to try at least to relieve any pathologic condition that might exist. The operation was done, the thickened portion of bone removed, and during convalescence the prisoner's character seemed to change. From being sullen and morose he became bright and cheerful, walked with firmer step, held his head erect and appeared to be a different man. It is not surprising then that a few months after the operation the governor was induced to set him free on parole, and there seemed to be every reason to hope that a useful citizen had been restored to society in place of a criminal that had been taken from it.

Unfortunately the arrest of the paroled prisoner during the first week in January of the present year, for a series of burglaries with regard to which the evidence is complete, seems to make it clear that the improvement was only temporary, or that the operation and his subsequent good conduct were steps in a scheme to secure his release from prison. The gang of burglars with which he has been working has been one of the most dangerous in western New York. Evidence seems to show that he was actually the leader and the inspiring genius of the gang. He has not been led astray into crime the second time, but he has actually been the perverter of others. It is just four years since his release, and for nearly a year he was under the surveilance of the police, who declared that he was leading an honest life. He seems to have been aware of their survillance and of the fact that it had ceased, for almost immediately afterward began the series of burglaries which has finally landed him in prison again.

This result or something similar is of course no more than what might have been expected. There is no trustworthy evidence to show that perversions of moral character, independent or mental deterioration, result from pressure on the brain. During the Civil War a large number of injuries to the head by penetrating wounds of various kinds with subsequent recovery were noted, yet perversions of character followed in so few cases that they must be taken as representing coincidences and not consequences. Very severe injuries to the brain, as for instance in the famous crowbar case, have been followed by actual improvement of character rather than the reverse, although, of course, there is no more reason to trace a relation of cause and effect here than in the instances of deterioration. Without any injuries, men have been known to change from respected citizens to criminals or at least to violators of law. Even in cases of insanity following injuries to the head, moreover, favorable results from operative interference are very infrequent.

Sensational announcements of improvement in such cases after surgical intervention, like those that used to be made for various surgical procedures in epilepsy, need to be controlled by the subsequent history of the case. Immediate improvement in both sets of cases is usually rather mental than physical in origin, and while the possibility of improvement in certain severe cases justifies surgical intervention, successes reported before many years have tested their permanence are liable to produce false impressions.—J. A. M. A.

